# The Role of Glucagon-Like Peptide 1 (GLP1) in Type 3 Diabetes: GLP-1 Controls Insulin Resistance, Neuroinflammation and Neurogenesis in the Brain

**DOI:** 10.3390/ijms18112493

**Published:** 2017-11-22

**Authors:** Choon Sang Bae, Juhyun Song

**Affiliations:** Department of Anatomy, Chonnam National University Medical School, Gwangju 61469, Korea; csbae@chonnam.ac.kr

**Keywords:** glucagon like peptide 1 (GLP-1), type 3 diabetes, diabetes-induced dementia, Alzheimer’s disease (AD), insulin resistance, Amyloid beta (Aβ)

## Abstract

Alzheimer’s disease (AD), characterized by the aggregation of amyloid-β (Aβ) protein and neuroinflammation, is the most common neurodegenerative disease globally. Previous studies have reported that some AD patients show impaired glucose utilization in brain, leading to cognitive decline. Recently, diabetes-induced dementia has been called “type 3 diabetes”, based on features in common with those of type 2 diabetes and the progression of AD. Impaired glucose uptake and insulin resistance in the brain are important issues in type 3 diabetes, because these problems ultimately aggravate memory dysfunction in the brain. Glucagon-like peptide 1 (GLP-1) has been known to act as a critical controller of the glucose metabolism. Several studies have demonstrated that GLP-1 alleviates learning and memory dysfunction by enhancing the regulation of glucose in the AD brain. However, the specific actions of GLP-1 in the AD brain are not fully understood. Here, we review evidences related to the role of GLP-1 in type 3 diabetes.

## 1. Introduction

Alzheimer’s disease (AD) as an age-related neurodegenerative disorder is not well understood in terms of etiology, even though it was first described over 100 years ago [1]. AD is characterized by extracellular accumulation of aggregated amyloid-β (Aβ) protein, intracellular accumulation of hyper-phosphorylated tau protein, neuroinflammation, and a reduction in cerebral glucose consumption [2]. Recent studies have demonstrated that AD has a pathophysiological relationship with type 2 diabetes mellitus (T2DM), in that both involve impairment of insulin signaling and glucose metabolism [3]. Epidemiological studies have indicated that T2DM increases the risk of AD [4,5]. The brain has been known to regulate body energy and control food intake and body weight [6,7]. Additionally, the brain consumes glucose at a high rate, and uses it for propagation of action potentials and maintenance of the membrane potentials required for neuronal transmission [8,9]. AD patients show decreased glucose utilization in brain areas that are directly related to cognitive functions, including the hippocampus and cerebral cortex [10]. According to several studies, the deregulation of glucose metabolism in AD can be controlled by the administration of a hormone known as a potent regulator of glucose homeostasis [11] and of food intake [12], glucagon-like peptide 1 (GLP-1) [13]. The fact that administration of this peptide improves cognitive decline in patients with AD, as well as in AD mouse model [14,15] suggests that deregulation of glucose in the brain is a crucial issue in the onset and progression of AD [4,5,16,17,18]. Here, we review recent evidence concerning the role of GLP-1 in diabetes-induced dementia. We highlight the importance of GLP-1 in the onset and progression of diabetic AD, sometimes referred to as type 3 diabetes.

## 2. Diabetes Induced Dementia as the Type 3 Diabetes

Recent studies have demonstrated that patients with T2DM and metabolic syndrome have elevated risk for vascular dementia and AD [19,20]. Other studies have reported aberrant cerebral insulin homeostasis, which is called insulin resistance, in AD patients [21,22]. In the CNS, insulin is synthesized in neurons such as pyramidal and granule cells in the cerebral cortex and hippocampus [23,24]. Pancreatic insulin transported in small amounts across the blood–brain barrier (BBB) could also influence brain function [25,26]. Insulin growth factor-1 (IGF-1) and its receptor (IGF-1R) can be observed in the brain and have been related to the control of neurogenesis and synaptogenesis [27,28]. Deregulation of brain insulin signaling and IGF-1 signaling affects insulin resistance, energy metabolism, and lipid metabolism and results in pathological changes in the central nervous system (CNS) [29,30,31,32]. According to several studies, insulin and IGF-1 resistance can be detected in the brains of AD patients [29], but the relationship between insulin resistance and brain dysfunction remains unclear [33]. Recently, the relationship between brain insulin/IGF-1 signaling impairment and AD has been dubbed type 3 diabetes [34]. Further study of the mechanisms involved in the onset and progression of type 3 diabetes is necessary to improve our understanding of its pathology type 3 diabetes.

## 3. Glucagon-Like Peptide 1 (GLP1)

GLP-1 is an endogenous incretin hormone of 30-amino acids, produced by enteroendocrine L-cells, that influences food ingestion [35,36], enhances glucose-induced insulin secretion from pancreatic islets [37], and can act as a neuropeptide when released in the brain [38]. GLP-1 receptors (GLP-1R) exist widely throughout the brain, in areas including the hypothalamus, thalamus, hippocampus, cortex, and brainstem nucleus [39,40,41]. GLP-1 and other GLP-1 analogues can cross the BBB [42,43]. Because GLP-1 and its receptors exist in both the CNS and peripheral tissues, the effect of GLP-1 on energy metabolism is mediated by both the CNS and the peripheral nervous system (PNS) [11,44,45]. Moreover, GLP-1 is synthesized by neurons within the nucleus of the solitary tract [46,47]. These neurons have long projections to hypothalamic, thalamic, and cortical brain areas [48]. GLP-1 contributes to glycemic homeostasis and GLP1R agonists such as exendin-4, liraglutide, and lixisenatide have been approved to treat T2DM [49,50]. Furthermore, GLP-1 increases the spontaneous activity of neurons in the hippocampal CA1 region and promotes excitatory synaptic transmission in the hippocampus [51]. GLP-1 receptor knockout mice show decreased memory retention in the Morris water maze task, and the administration of GLP-1 agonists leads to improvement in learning and memory [52]. Here, given that GLP-1 could regulate glucose metabolism and potentially be used for treatment of T2DM [44,49], we focused on the role of GLP-1 in type 3 diabetes, highlighting the therapeutic importance of GLP-1 in diabetes-induced dementia.

## 4. The Effect of GLP-1 in Type 3 Diabetes: GLP-1 Attenuates Neuroinflammation and Improves Neurogenesis and Insulin Sensitivity in AD

One study suggested that GLP-1 mimetic drugs have neuroprotective, neurotrophic, and anti-inflammatory effects, which play a role in retardation of AD progression [14]. Another study demonstrated that liraglutide, a GLP-1 receptor agonist, can alleviate spatial memory dysfunction and neuroinflammation that leads to cognitive impairment [53]. GLP1 has been shown to act as a growth factor in the brain and promote neurite growth [54]. GLP-1 receptor activators stimulate the differentiation of neuronal stem cells in a manner similar to nerve growth factor, so it may inhibit brain atrophy in AD patients [55]. Additionally, GLP1 receptor agonists such as liraglutide and exendin-4 attenuate endogenous levels of amyloid beta in the brain and prevent amyloid plaque accumulation in the AD brain [42,53]. Furthermore, stimulating glucose metabolism in AD patients through the administration of GLP-1 markedly improves cognitive dysfunction in the AD brain [56,57]. In APP/PS1 mice (a mouse model of AD) brain, liraglutide and GLP-1 increase long-term potention (LTP) [42,58] and increase synaptic plasticity [41,55,59]. Moreover, GLP-1 has been found to improve insulin sensitivity [60,61] and control energy metabolism [62,63]. Recent studies reported that GLP-1 could attenuate brain insulin resistance by decreasing c-Jun N-terminal kinase (JNK) signaling and increasing the expression of the B-cell lymphoma 2 gene (*Bcl2*) in the T2DM mouse [64]. One study demonstrated that liraglutide treatment in an AD mouse model triggers the activation of microglia in the brain [42]. Neurogenesis, the generation of new neurons from neuronal progenitor stem cells [65,66], occurs in the subventricular zone (SVZ) of the lateral ventricles and the subgranular zone of the hippocampal [67,68]. According to previous results, adult neurogenesis is linked to memory function and the facilitation of LTP [69,70]. In the AD brain, a decrease in neurogenesis is commonly observed and aggravates the disease pathology [41,71]. Several studies found that GLP-1 receptor agonists increase the proliferation of neural progenitor cells [41] and increase neurogenesis in the dentate gyrus of the hippocampus [43]. Earlier studies reported the impaired proliferation of neural stem cell in the AD mouse model [66,72] and that GLP-1 and analogues of GLP-1 can promote neural stem cell proliferation in the brain [73,74]. GLP-1 receptor activates neurogenesis in hippocampus through mitogen activated protein kinases (MAPK) [75], leading to enhancement of learning and memory [75,76,77]. Collectively, GLP-1 could attenuate neuroinflammation and enhance neurogenesis and insulin resistance in diabetes-induced dementia, also known as type 3 diabetes.

## 5. Conclusions

Summing up, we suggest that GLP-1 is a good candidate for improving cognitive dysfunction in diabetes-induced dementia. First, GLP-1 could attenuate the inflammatory responses in brain caused by amyloid beta (Aβ)-induced oxidative stress. GLP-1 could regulate the activation of microglia and protect neurons against oxidative stress. Second, GLP-1 could promote neurogenesis in AD brain. This means that GLP-1 could stimulate the generation of new neurons to replace damaged neurons in the AD brain. Finally, GLP-1 can alleviate insulin resistance in the AD brain, suggesting that impaired glucose metabolism and insulin resistance leads to severe memory dysfunction. To conclude, our study highlights that manipulation of GLP-1 may be an effective therapy for improving AD-like pathology in diabetes-induced dementia, also known as type 3 diabetes.
